# External validation of two prediction models identifying employees at risk of high sickness absence: cohort study with 1-year follow-up

**DOI:** 10.1186/1471-2458-13-105

**Published:** 2013-02-05

**Authors:** Corné AM Roelen, Ute Bültmann, Willem van Rhenen, Jac JL van der Klink, Jos WR Twisk, Martijn W Heymans

**Affiliations:** 1 365/Occupational Health Service, PO Box 85091, 3508 AB, Utrecht, the Netherlands; 2 Department of Health Sciences section Methodology and Applied Biostatistics, VU University, De Boelelaan 1085-1087, 1081 HV, Amsterdam, the Netherlands; 3 Department of Health Sciences section Community and Occupational Medicine, University Medical Center Groningen, University of Groningen, PO Box 196, 9700 AD, Groningen, the Netherlands; 4 Center for Human Resource, Organization and Management Effectiveness, Business University Nyenrode, PO Box 130, 3620 AC, Breukelen, the Netherlands

**Keywords:** Absenteeism, Forecasting, Generalization, Office workers, Regression prognostics, Sick leave, Transportability

## Abstract

**Background:**

Two models including age, self-rated health (SRH) and prior sickness absence (SA) were found to predict high SA in health care workers. The present study externally validated these prediction models in a population of office workers and investigated the effect of adding gender as a predictor.

**Methods:**

SRH was assessed at baseline in a convenience sample of office workers. Age, gender and prior SA were retrieved from an occupational health service register. Two pre-defined prediction models were externally validated: a model identifying employees with high (i.e. ≥30) SA days and a model identifying employees with high (i.e. ≥3) SA episodes during 1-year follow-up. Calibration was investigated by plotting the predicted and observed probabilities and calculating the calibration slope. Discrimination was examined by receiver operating characteristic (ROC) analysis and the area under the ROC-curve (AUC).

**Results:**

A total of 593 office workers had complete data and were eligible for analysis. Although the SA days model showed acceptable calibration (slope = 0.89), it poorly discriminated office workers with high SA days from those without high SA days (AUC = 0.65; 95% CI 0.58–0.71). The SA episodes model showed acceptable discrimination (AUC = 0.76, 95% CI 0.70–0.82) and calibration (slope = 0.96). The prognostic performance of the prediction models did not improve in the population of office workers after adding gender.

**Conclusion:**

The SA episodes model accurately predicted the risk of high SA episodes in office workers, but needs further multisite validation and requires a simpler presentation format before it can be used to select high-risk employees for interventions to prevent or reduce SA.

## Background

Sickness absence (SA) is an indicator of the health status of working populations [1-4]. Long-term SA not only reflects poor health, but also excludes individuals from the labor market and restricts social participation. The chances of getting back to work decrease with increasing SA duration [5,6]. Hence, it is of great importance to prevent long-term SA and pay attention to employees still at work, but at high risk of long-term SA. The importance of identifying employees at risk of long-term SA is further underlined by randomised-controlled trials showing that preventive consultations reduced the number of SA days in high-risk employees [7-9], but were not cost-effective in employees with moderate or low SA risks [9].

Questionnaires have been developed to identify employees with a high SA risk [7-13]. However, questionnaire surveys often have moderate response rates and healthy employees are more likely to participate in surveys than employees with health problems, known as the ‘healthy volunteer effect’ [14-16]. Hence, employees at risk of SA may be missed in questionnaire surveys due to selective non-response [16]. A prediction model or rule that includes readily available factors, would be practical for physicians to identify employees at risk of high SA. Although not all employees visit physicians or other health care providers, they will be more likely to be at risk of high SA than the ‘healthy volunteers’ participating in questionnaire surveys.

Recently, two prediction models including age, prior SA and self-rated health (SRH) were developed in a sample of 535 health care workers [17]. The SA days model ln(odds)_SA_ = 0.601–0.016*age + 0.007*prior SA–0.718*SRH, fairly discriminated health care workers with high SA days from those with few SA days and showed acceptable calibration i.e., adequate agreement between predicted and observed probabilities of high SA days. The SA episodes model ln(odds)_SA_ = 0.806–0.043*age + 0.472*prior SA–0.715*SRH showed good discrimination and calibration. Although both models were internally validated by bootstrapping techniques, they were not yet tested in another working population. Internally validated prediction models may degrade in other populations due to under- or overfitting [18,19]. Underfitting occurs when important predictors of high SA are missing and overfitting occurs when a prediction model is too much adapted to the dataset in which it was developed.

Hence, the ability of the SA prediction models to provide accurate predictions in other workers still needs to be addressed. McGinn et al. defined four levels of validation for prediction models. The fourth and lowest level is the development and internal validation of a prediction model. The third level represents validation in another small sample and the second level is reached by validation in large samples or multiple settings. The first and highest level is achieved when the use of a prediction model leads to a change in decision-making in medical practice and improvement of patients outcomes [20].

To further develop the SA prediction models to a higher level, the present study externally validated these models, which were developed in a population of health care workers, in a new population of office workers. The following research questions were addressed:

i) How is the external predictive performance of the SA days model in office workers?

ii) How is the external predictive performance of the SA episodes model in office workers?

The previous development setting was a female-dominated population of health care workers and, therefore, gender was discarded at the time of development. However, gender is an obvious characteristic to consider for the prediction of SA [21]. Women are more frequently absent from work than men, possibly because of gender-related organizational and psychosocial work characteristics or gender differences in work-related factors interacting with person-related factors in family life [22]. Therefore, the present study re-estimated the prediction models in office workers and evaluated the effect of adding gender.

## Methods

### Study population and setting

In November 2006, 1,137 office workers of an insurance company were invited to participate in an occupational health check-up. The health check-up questionnaire assessed general health, mental health, work conditions, and the working environment. General health was investigated with the question: *In general, how would you rate your health*? This question has been used as a health measure in surveys worldwide and was found to be associated with various morbidity measures and the use of health services [23]. For SRH, office workers rated their health in categories 4 = “excellent”, 3 = “good”, 2 = “fair” and 1 = “poor”. The Medical Ethics Committee of the University Medical Center Groningen granted ethical clearance for linking the health check-up data to the SA data.

### Sickness absence data

Sickness absence (SA) was defined as absence from work due to work-related and non work-related injuries or illnesses. SA data were retrieved from an occupational health service register that records SA from the day of reporting sick to the day an employee resumed work at equal earnings as before SA. The calendar days between the first and last SA day were accumulated. For example, if a worker is off work 1 day on one occasion and 5 days on another, this was counted as 6 SA days and 2 SA episodes, unless the episodes were less than 28 days apart. In line with Dutch SA insurance policies, SA episodes with less than 28 days worked between them were regarded as one SA episode. The total number of SA days in 2005 and 2006 was tallied for each employee as a measure for prior SA in the SA days model. Likewise, the total number of SA episodes in 2005 and 2006 was accumulated for each employee as a measure for prior SA in the SA episodes model.

The number of SA days and episodes were also recorded for each employee during 1-year follow-up in 2007. At the development of the prediction models, high SA days was defined as ≥30 accumulated (not necessarily consecutive) SA days and high SA episodes as ≥3 episodes during 1-year follow-up [17]. The same definitions for high SA were adopted in the current external validation study.

### External validation of prediction models

The original prediction models were applied in the external dataset with fixed regression coefficients i.e., by transporting the regression coefficients from the development setting to the validation setting. The external validity of predictions was quantified by performance measures related to discrimination and calibration [24-26]. Discrimination was evaluated by the area under the receiver operating characteristic curve (AUC). An AUC of 0.5 indicates no discrimination above chance and an AUC of 1.0 indicates perfect discrimination. Generally, an AUC = 0.9-1.0 represents excellent, AUC = 0.8-0.9 good, AUC = 0.7-0.8 fair, and AUC = 0.6-0.7 poor discriminative ability. Discrimination is assumed to be useful if AUC ≥0.75 [27]. The prognostic validity of the prediction models was investigated in more detail by calculating the sensitivity and specificity at different cut-off points of predicted high SA probabilities. Calibration was assessed by plotting predicted probabilities with fixed regression coefficients obtained from the development setting of health care workers against the observed probabilities in the population of office workers [24-26]. Calibration was expressed in the calibration slope with a calibration slope = 1 indicating perfect calibration.

### Updating the prediction models

The aforementioned external validation kept the regression coefficients fixed at their original value obtained from the development setting. Updating was performed by model revision, which is the re-estimation of the regression coefficients of predictor variables and/or considering more covariates for inclusion in the model [25,26,28]. First, the regression coefficients of the prediction models were re-estimated for the population of office workers. After re-estimation of the regression coefficients, gender was added as a predictor to the prediction models. The effect of including gender was assessed by using the Likelihood Ratio (LR) test and significance was concluded for LR p < 0.05. Furthermore, we evaluated the effect of excluding SRH from the prediction models, since SRH is not usually recorded in SA registers. The effect of excluding SRH was also assessed by LR testing.

### Software

External validation was performed in R (R Development Core Team, 2009) using Harrell’s Regression Modeling Strategies (rms) package, version 3.2-0 [29].

## Results

A total of 633 office workers (56%) participated in the health check-ups. Participants had a mean age of 44.5 (standard deviation [SD] = 9.3) years and non-participants 39.0 (SD = 9.4) years (t-test for independent samples p < 0.01). Of the participants, 62% were men as compared to 68% of non-participants (Chi-square p = 0.04). Participants had fewer SA episodes (Chi-square p < 0.01) than non-participants; 15% of participants had high SA episodes as compared to 22% of non-participants (Chi-square p < 0.01). Categories of SA days (Chi-square p = 0.16) and the proportions of high SA days (Chi-square p = 0.45) did not differ between participants and non-participants.

Among participants, the response on SRH was missing in 5 cases and SA data were missing in the occupational health service register in another 35 cases. These 40 workers (6%) with missing data were excluded from analysis. Hence, the study population for external validation consisted of 593 office workers with complete data (Table 1).

**Table 1:** Study population characteristics (N = 593)

**Characteristics**	
Gender, n (%)	
women	223 (38%)
men	370 (62%)
Self-rated health, n (%)	
excellent	149 (25%)
good	337 (57%)
fair	82 (14%)
poor	25 (4%)
Prior sickness absence days, n (%)	
0	123 (20%)
1-10	195 (33%)
11-29	107 (18%)
30-60	110 (19%)
>60	58 (10%)
Prior sickness absence episodes, n (%)	
0	123 (21%)
1	132 (22%)
2	118 (20%)
3	77 (13%)
4	64 (11%)
≥5	79 (13%)

### External validation of prediction models

**Figure 1:**
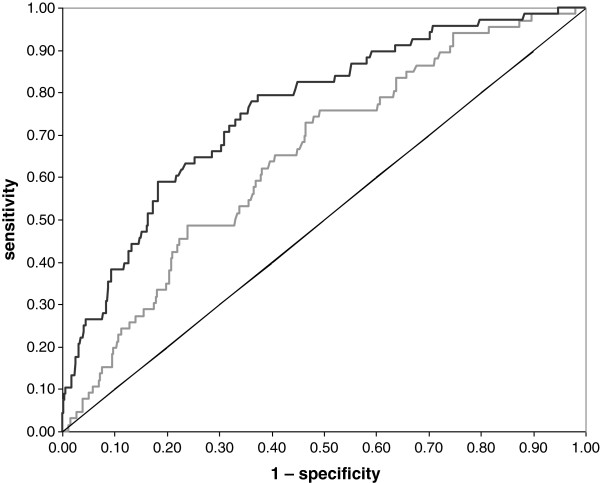
**Discriminative ability at external validation.** The figure shows the ROC curves of the models identifying office workers with high sickness absence days (grey line; AUC = 0.65 with 95% CI = 0.58 – 0.71) and high sickness absence episodes (black line; AUC = 0.76 with 95% CI = 0.70 – 0.82); the diagonal indicates no discrimination above chance.

A total of 66 (11%) office workers had high SA days and 67 (11%) office workers had high SA episodes during follow-up; 29 office workers had both high SA days and episodes. Figure 1 shows the receiver operating characteristic curves, which reflected a poor discriminative ability of the SA days model (AUC = 0.65; 95% CI = 0.58 – 0.71) and a fair discriminative ability of the SA episodes model (AUC = 0.76; 95% CI = 0.70 – 0.82). The sensitivity and specificity at different cut off points for the probability of high SA episodes are shown in Table 2.

**Table 2:** Prognostic characteristics of the episodes model at external validation

**Cut off probability**	**N**	**sens**^**a**^	**spec**^**b**^	**PPV**^**c**^	**NPV**^**d**^
≥10%	251	0.79	0.62	0.22	0.96
≥20%	139	0.59	0.81	0.29	0.94
≥30%	85	0.38	0.89	0.31	0.91
≥40%	60	0.28	0.92	0.32	0.91
≥50%	33	0.22	0.97	0.45	0.91
≥60%	20	0.13	0.98	0.45	0.90
≥70%	9	0.09	0.99	0.67	0.89
≥80%	8	0.09	1.00	0.75	0.89
≥90%	4	0.04	1.00	0.75	0.88

^a^ sensitivity; ^b^ specificity; ^c^ positive predictive value; ^d^ negative predictive value.

The table shows the prognostic characteristics for each cut-off SA probability of the model identifying office workers (N = 593) with high SA episodes at external validation with fixed coefficients.

**Figure 2:**
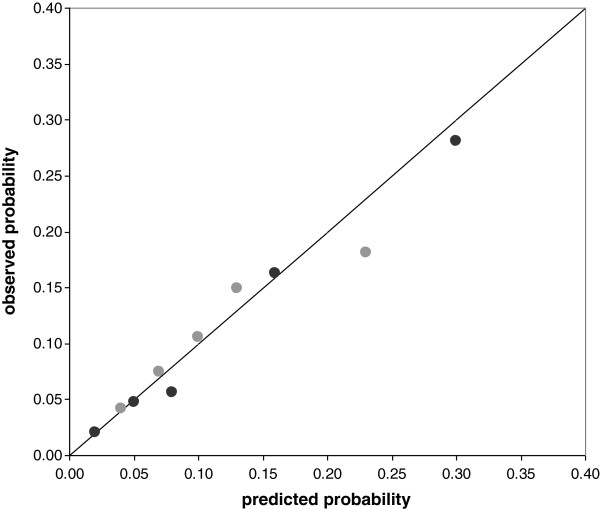
**Calibration plot.** The figure shows probabilities of high SA predicted by the SA days model (grey dots) and the SA episodes model (black dots) with fixed regression coefficients from the development setting, and the observed probabilities of high SA in office workers per quintile of predicted probabilities; the diagonal indicates perfect calibration.

Calibration was acceptable for both prediction models, as is shown in the calibration plot (Figure 2) with calibration slopes of 0.89 for the SA days model and 0.96 for the SA episodes model.

### Updating the prediction models

Re-estimation of the regression coefficients of the SA days model showed that the regression coefficients of prior SA days and SRH in office workers were half the values of the development sample. The SA days model improved after gender was added (LR-test p < 0.01), but its predictive performance was similar to that of the SA days model without gender (Table 3). The SA days model significantly degraded (LR-test p = 0.04) when SRH was excluded.

**Table 3:** Performance of sickness absence (SA) prediction models

	**Development setting**	**Validation setting**			
		**Fixed coefficients**	**Re-estimated coefficients**	**Gender inclusive**	**SRH exclusive**
**SA days model**					
Regression coefficients (SE^a^)					
Age	−0.016 (0.015)	−0.016 (0.015)	−0.016 (0.014)	0.004 (0.014)	−0.001 (0.014)
Prior SA	0.007 (0.001)	0.007 (0.001)	0.003 (0.002)	0.003 (0.002)	0.004 (0.002)
Self-rated health	−0.718 (0.244)	−0.718 (0.244)	−0.356 (0.170)	−0.349 (0.173)	not included
Gender	not included	not included	not included	0.699 (0.269)	not included
Predictive performance					
Nagelkerke’s pseudo R^2^	0.12	0.03	0.03	0.05	0.02
Discrimination (AUC^b^)	0.73	0.65	0.68	0.68	0.65
Calibration (slope)	0.94	0.89	0.87	0.86	0.86
**SA episodes model**					
Regression coefficients (SE^a^)					
Age	−0.043 (0.016)	−0.043 (0.016)	−0.039 (0.015)	0.008 (0.015)	0.005 (0.015)
Prior SA	0.472 (0.070)	0.472 (0.070)	0.465 (0.067)	0.473 (0.068)	0.477 (0.065)
Self-rated health	−0.715 (0.255)	−0.715 (0.255)	−0.190 (0.185)	−0.187 (0.188)	not included
Gender	not included	not included	not included	0.463 (0.256	not included
Predictive performance					
Nagelkerke’s pseudo R^2^	0.32	0.18	0.21	0.22	0.21
Discrimination (AUC^b^)	0.83	0.76	0.78	0.78	0.77
Calibration (slope)	0.98	0.96	0.98	0.95	0.98

^a^ standard error; ^b^area under the receiver operating characteristic curve.

The table shows the regression coefficients and performance measures in a development sample of 535 health care workers and the current validation sample of 593 office workers.

With regard to the SA episodes model, the re-estimated regression coefficient of SRH was reduced from −0.715 at the development setting to −0.190 at the current validation setting. The SA episodes model neither improved after adding gender (LR-test p = 0.11), nor degraded after excluding SRH (LR-test p = 0.31).

## Discussion

The SA days model poorly discriminated between office workers with and without high SA days, whereas the SA episodes model showed fair discrimination and acceptable calibration. Although gender was associated with SA, particularly SA days, the predictive performance of the models did not improve after adding gender. It would have been interesting to add other readily available work-related or person-related variables from the health check-up, but the number of high SA events restricted the number of predictors in the SA prediction models. Generally, it is advised to include one predictor per 15 or more events [25]. With an effective sample size of 66 employees with high SA days and 67 employees with high SA episodes, the prediction models could only include four predictors in the present validation setting.

Although SRH is easy to obtain without the need for questionnaire surveys, employees have to be asked to rate their health. Thus, SRH can only be gathered at worksite health fairs or from employee visits to health care departments. Our study showed that the predictive performance of the SA episodes model was maintained after deleting SRH from the prediction model. This implicates that age and prior SA, which are regular SA register data, would suffice to identify white collar worker at risk of high SA episodes. However, it should be noted that SRH was a stronger predictor in the health care setting where the prediction models were developed. Excluding strong predictors considerably reduces the predictive ability of prediction models. Thus, if available, SRH should be included in the SA episodes model, because SRH is a health measure and SA is, at least partly, a health-related phenomenon.

### Prognostic performance

The discriminative ability of both prediction models degraded in the population of office workers, although the SA episodes model still showed fair performance. Furthermore, the cut-off probabilities of the SA episodes model confirm those of the development setting. At a cut-off risk of high SA of 10%, the sensitivity was acceptable, but the specificity was low due to high false-positive rates. A sensitive cut-off point can be used to identify as much office workers at risk of high SA as possible. For example, workers with high SA episodes may suffer chronic recurrent conditions that are not yet diagnosed or treated. From a societal perspective, it may be desirable to select workers with a ≥10% probability of high SA episodes for further diagnosis and treatment to prevent worsening of chronic conditions, long-term SA and subsequent disability pensioning. Alternatively, more specific cut-off points can be used to reduce false-positive rates, for instance to select high-risk office workers for costly interventions.

### Why did the prognostic performance degrade?

The purpose of a prediction model is to provide valid predictions for new subjects [24-26]. External validation refers to the transportability of a prediction model to other settings than where the model was developed [18,30]. Prediction models tend to perform better in the subjects used to develop the model than in other subjects, a phenomenon known as over-optimism [19]. For internal model validation, bootstrapping methods are recommended to provide bias-corrected estimates of model performance. In the development sample of health care workers, internal validation by bootstrapping revealed an over-optimism of 0.06 for the SA days model and 0.03 for the SA episodes model. Subsequently, the performance parameters were shrunken to adjust for this over-optimism [24-26,28]. Although adjustment for over-optimism by bootstrap techniques may not be sufficient in relatively small data sets [31], this low over-optimism made it unlikely that the poorer performance of the prediction models in the sample of office workers was due to overfitting to the development sample.

Alternatively, underfitting occurs when important predictors are missing from the prediction models. Internal validation by bootstrapping techniques will not detect underfitting because the bootstrap samples are drawn from the same population. The poorer performance of the prediction models in the present study may well be explained by underfitting, in particular because the Nagelkerke pseudo R^2^ values were lower than in the development sample of health care workers. The Nagelkerke’s pseudo R^2^ reflects the variance in high SA between office workers that is explained by the covariates fitted in the prediction models [32]. Low Nagelkerke’s pseudo R^2^ values indicate that other factors than those included in the model may be important for predicting high SA among office workers. Hence, future studies should further update the prediction models with other predictors, e.g. work variables and personal characteristics, provided that these variables are readily available or easy to obtain by physicians.

Another explanation for the lower performance may be the different case-mix in the population of office workers. Case-mix refers to the distribution of known and unknown predictors of SA in the studied populations. The population in which the prediction models were developed consisted of 535 health care workers, predominantly female nurses who were younger than the office workers in the present study. One-third of the development population of health care workers reported excellent health as compared to a quarter of the present population of office workers. Furthermore, 8% of healthcare workers reported less than good health as compared to 18% of office workers. The distribution of prior SA did not differ between the development and the validation populations.

Finally, the regression coefficients may *really* differ between the two working populations i.e., the working populations were not plausibly related. The prediction models were developed in health care workers, predominantly working in physically and emotionally demanding nursing care. Possibly, this development sample differed too much from the current validation sample of office workers performing mentally demanding work at an insurance company. Furthermore, the ‘healthy worker effect’, which selects the healthiest employees to work until older age, may be greater in nursing care which is more physically demanding than office work. This may explain why the inverse association between age and high SA was stronger in the development sample of health care workers than in the validation sample of office workers. The ‘healthy worker effect’ may also explain why SRH was a stronger predictor of high SA in health care workers than in office workers, particularly since SRH was found to reflect physical functioning rather than mental health [33].

### Practical implications and future directions

Prediction models have practical perspectives if they accurately predict outcomes for different populations [18,30]. This study showed that the SA episodes model accurately predicted the risk of high SA episodes in both health care workers and office workers. Therefore, this prediction model may be a promising tool to select employees at risk of high SA episodes for preventive occupational health consultations. Such consultations were found to reduce SA duration [7,8], but not SA frequency [7]. Duijts et al. reported that in employees who received preventive coaching the mean SA duration was 11.7 days during 8 – 12 months follow-up as compared to 13.1 days in the control group. The mean SA frequencies were 1.07 and 1.40 respectively, though none of the differences in SA measures was statistically significant [34]. In the current study, the SA episodes model identified employees at risk of a high SA frequency, but the model may also indirectly identify employees at risk of future long SA duration, because frequent SA has been recognized as a risk factor for long-term SA [35-37]. Further research is needed to clarify which frequent absentees develop long-term SA in the future.

It is also important to further validate the SA episodes model, for example in large heterogeneous populations and in multiple settings [18,20]. The more numerous and diverse the settings in which the SA episodes model accurately predicts high SA, the more likely it will generalize to untested working populations [18]. Furthermore, the SA episodes model should be developed into a nomogram or score chart that is easier to understand and use in daily practice than the regression formula. Simpler presentation formats provide approximate predictions, but this will not be problematic for identifying employees at risk of high SA.

## Conclusions

Although the SA days model showed acceptable calibration, it poorly discriminated office workers with high SA from those without high SA. The SA days model was probably underfitted and needs updating by adding predictors of SA duration. The SA episodes model accurately predicted the risk of high SA among office workers, but needs further multisite validation and requires a simpler presentation format before it can be used to select high-risk employees for interventions to prevent or reduce frequent SA.

## Competing interests

The authors declare that they have no competing interests.

## Authors’ contributions

CAMR conceived and designed the study, retrieved, analyzed, and interpreted the data, and drafted the manuscript. UB interpreted the data and drafted the manuscript. WvR, JJLvdK and JWRT revised the manuscript critically for its intellectual content. MWH analyzed and interpreted the data. All authors have read and approved the final version of the manuscript.

## Pre-publication history

The pre-publication history for this paper can be accessed here:

http://www.biomedcentral.com/1471-2458/13/105/prepub

## References

[B1] Marmot M Feeney A Shipley M North F Syme SL Sickness absence as a measure of health status and functioning: from the UK Whitehall II study J Epidemiol Community Health 1995 49 124 130 10.1136/jech.49.2.124 7798038 PMC1060095

[B2] Kivimäki M Head J Ferrie JE Shipley MJ Vahtera J Marmot MG Sickness absence as a global measure of health: evidence from mortality in the Whitehall II prospective cohort study BMJ 2003 327 364 368 10.1136/bmj.327.7411.364 12919985 PMC175810

[B3] Bambra C Norman P What is the association between sickness absence, morbidity and mortality? Health Place 2006 12 728 733 10.1016/j.healthplace.2005.02.008 16814198

[B4] Eriksson HG von Celsing HS Wahlström R Janson L Zander V Wallman T Sickness absence and self-reported health a population-based study of 43,600 individuals in central Sweden BMC Publ Health 2008 8 426 10.1186/1471-2458-8-426 PMC2627845 19116000

[B5] Labriola M Conceptual framework of sickness absence and return to work, focusing on both the individual and contextual level Work 2008 30 377 387 18725701

[B6] Lund T Kivimäki M Labriola M Villadsen E Christensen KB Using administrative sickness absence data as a marker of future disability pension: the prospective DREAM study of Danish private sector employees Occup Environ Med 2008 65 28 31 10.1136/oem.2006.031393 17626139

[B7] Kant IJ Jansen NW van Amelsvoort LG van Leusden R Berkouwer A Structured early consultation with the occupational physician reduces sickness absence among office workers at high risk for long-term sickness absence: a randomized controlled trial J Occup Rehabil 2008 18 79 86 10.1007/s10926-007-9114-z 18196446 PMC2668565

[B8] Taimela S Malmivaara A Justén S Läärä E Sintonen H Tiekso J Aro T The effectiveness of two occupational health intervention programmes in reducing sickness absence among employees at risk Two randomised controlled trials. Occup Environ Med 2008 65 236 241 10.1136/oem.2007.032706 PMC2564865 17681994

[B9] Taimela S Justén S Aronen P Sintonen H Läärä A Malmivaara A Tiekso J Aro T An occupational health intervention programme for workers at high risk for sickness absence. Cost effectiveness analysis based on a randomised controlled trial Occup Environ Med 2008 65 242 248 10.1136/oem.2007.033167 17933885 PMC2564864

[B10] Duijts SF Kant IJ Landeweerd JA Swaen GMH Prediction of sickness absence: development of a screening instrument Occup Environ Med 2006 63 564 569 10.1136/oem.2005.024521 16698807 PMC2078122

[B11] Roelen CA van der Pol TR Koopmans PC Groothoff JW Identifying workers at risk of sickness absence by questionnaire Occup Med 2006 56 442 446 10.1093/occmed/kql087 16905625

[B12] Duijts SF Kant I van den Brandt PA Swaen GM Psychometrics and validation of a screening instrument for sickness absence Occup Med 2008 58 413 418 10.1093/occmed/kqn070 18567614

[B13] Kant IJ Jansen NW van Amelsvoort LG Swaen GM van Leusden R Berkouwer A Screening questionnaire Balansmeter proved successful in predicting future long-term sickness absence in office workers J Clin Epidemiol 2009 62 408 414 10.1016/j.jclinepi.2008.07.003 18986798

[B14] Etter JF Perneger TV Analysis of non-response bias in a mailed health survey J Clin Epidemiol 1997 50 1123 1128 10.1016/S0895-4356(97)00166-2 9368520

[B15] Froom P Melamed S Kristahl-Boneh E Benbassat J Ribak J Healthy volunteer effect in industrial workers J Clin Epidemiol 1999 52 731 735 10.1016/S0895-4356(99)00070-0 10465317

[B16] Duijts SF Kant IJ Swaen GM Advantages and disadvantages of an objective selection process for early intervention in employees at risk for sickness absence BMC Publ Health 2007 7 67 10.1186/1471-2458-7-67 PMC1868720 17474980

[B17] Roelen CA van Rhenen W Bültmann U Groothoff JW van der Klink JJ Heymans MW The development and validation of two prediction models to identify employees with high sickness absence Eur J Public Health 2013 23 128 133 10.1093/eurpub/cks036 22539631

[B18] Justice AC Covinsky KE Berlin JA Assessing the generalizability of prognostic information Ann Intern Med 1999 30 515 524 10075620 10.7326/0003-4819-130-6-199903160-00016

[B19] Babyak MA What you see may not be what you get: a brief nontechnical introduction to overfitting in regression-type models Psychosom Med 2004 66 411 421 10.1097/01.psy.0000127692.23278.a9 15184705

[B20] McGinn TG Guyatt GH Wyer PC Naylor CD Stiell IG Richardson WS Users’ guide to the medical literature XXII: How to use articles about clinical decision rules J Am Med Assoc 2000 284 79 84 10.1001/jama.284.1.79 10872017

[B21] Allebeck P Mastekaasa A Risk factors for sick leave – general studies Scand J Public Health 2004 32 suppl63 49 108 10.1080/14034950410021853 15513654

[B22] Bekker MH Rutte CG van Rijswijk K Sickness absence: a gender focused review Psychol Health Med 2009 14 405 418 10.1080/13548500903012830 19697251

[B23] Bowling A Just one question: if one question works, why ask several? J Epidemiol Community Health 2005 59 342 345 10.1136/jech.2004.021204 15831678 PMC1733095

[B24] Harrell FE Regression modelling strategies: applications to linear models, logistic regression, and survival analysis 2001 New York: Springer

[B25] Steyerberg EW Clinical prediction models 2009 New York: Springer

[B26] Steyerberg EW Vickers AJ Cook NR Gerds T Gonen M Obuchowski N Pencina NJ Kattan MW Assessing the performance of prediction models Epidemiology 2010 21 128 138 10.1097/EDE.0b013e3181c30fb2 20010215 PMC3575184

[B27] Fad J Upadhye S Worster A Understanding receiver operating characteristic (ROC) curves Can J Emerg Med 2006 8 19 20 10.1017/s1481803500013336 17175625

[B28] Steyerberg EW Borsboom GJ van Houwelingen HC Eijkemans MJ Habbema JD Validation and updating of predictive logistic regression models: a study on sample size and shrinkage Stat Med 2004 23 2567 2586 10.1002/sim.1844 15287085

[B29] Regression modelling strategieshttp://cran.r-project.org/web/packages/rms

[B30] Terrin N Schmid CH Griffith JL D’Agostino RB Selker HP External validity of predictive models: a comparison of logistic regression, classification trees, and neural networks J Clin Epidemiol 2003 56 721 729 10.1016/S0895-4356(03)00120-3 12954463

[B31] Bleeker SE Moll HA Steyerberg EW Donders AR Derksen-Lubsen G Grobbee DE Moons KE External validation is necessary in prediction research: a clinical example J Clin Epidemiol 2003 56 826 832 10.1016/S0895-4356(03)00207-5 14505766

[B32] Heinzl H Waldhör T Mittlböck M Careful use of pseudo R-squared measures in epidemiological studies Stat Med 2005 24 2867 2872 10.1002/sim.2168 16134131

[B33] Mavaddat N Kinmonth AL Sanderson S Surtees P Bingham S Khaw KT What determines self-rated health (SRH)? A cross-sectional study of SF-36 health domains in the EPIC-Norfolk Cohort J Epidemiol Community Health 2010 65 800 806 20551149 10.1136/jech.2009.090845

[B34] Duijts SF Kant I van den Brandt PA Swaen GM Effectiveness of a preventive coaching intervention for employees at risk for sickness absence due to psychosocial health complaints: results of a randomized controlled trial J Occup Environ Med 2008 50 765 776 10.1097/JOM.0b013e3181651584 18617832

[B35] Virtanen M Kivimäki M Vahtera J Elovainio M Sund R Virtanen P Ferrie JE Sickness absence as a risk factor for job termination, unemployment, and disability pension among temporary and permanent employees Occup Environ Med 2006 63 212 217 10.1136/oem.2005.020297 16497865 PMC2078149

[B36] Koopmans PC Roelen CA Groothoff JW Frequent and long-term sickness absence as a risk factor for work disability and job termination among employees in the private sector Occup Environ Med 2008 65 494 499 10.1136/oem.2007.034322 18032531

[B37] Koopmans PC Roelen CAM Groothoff JW Risk of future sickness absence in frequent and long-term absentees Occup Med 2008 58 268 274 10.1093/occmed/kqn040 18390880

